# Network Pharmacology and Serum Nontargeted Metabolomics Reveal the Protective Effects of Propionate Against Liver Damage Induced by a High-Fat and AGE-Rich Diet in Diabetic Mice

**DOI:** 10.1155/jdr/3955893

**Published:** 2025-07-02

**Authors:** Liang Wu, Jiajun Tan, Wen Sun, Xueyun Dong, Jiayuan He, Asmaa Ali, Min Chen, Leilei Zhang, Pingping Wang

**Affiliations:** ^1^Department of Laboratory Medicine, School of Medicine, Jiangsu University, Zhenjiang, China; ^2^Department of Laboratory Medicine, Taizhou Second People's Hospital, Taizhou, China; ^3^Jurong Hospital Affiliated to Jiangsu University, Zhenjiang, China; ^4^Health Testing Center, Zhenjiang Center for Disease Control and Prevention, Zhenjiang, China; ^5^Department of Pulmonary Medicine, Abbassia Chest Hospital, EMOH, Cairo, Egypt; ^6^Public Experiment and Service Center, Jiangsu University, Zhenjiang, China

**Keywords:** autophagy, diabetic liver injury, network pharmacology, nontargeted metabolomics, propionate

## Abstract

Diabetic liver injury is a leading cause of mortality in diabetes, with no specific treatment available. Sodium propionate (NaP) has anti-inflammatory and antioxidant properties, but its effectiveness in treating diabetic liver injury is still lacking research. The study employed network pharmacology to identify potential targets of NaP for Type 2 diabetes treatment, using oleic acid (OA) and advanced glycation end products (AGEs) to induce diabetic liver injury in HepG2 cells *in vitro*. Post-NaP intervention, Oil Red O staining assessed cellular lipid deposition, while Western blotting analyzed protein expression associated with oxidative stress, autophagy, and bile acid synthesis. NaP was administered to mice with diabetic liver injury induced by a high-fat and AGEs diet, and qPCR analysis was conducted to assess the expression of genes associated with inflammation, oxidative stress, and bile acid synthesis in the liver. HE staining was used to observe the liver injury, and nontargeted metabolomics analysis was used to analyze the effect of NaP on serum metabolic pathways. Network pharmacology analysis showed that NaP has anti-inflammatory and antioxidant effects, mainly involving multiple targets such as mitochondrial function, insulin resistance, and glucose metabolism. Experimental results in cells and animals demonstrated that NaP reduces lipid accumulation and inflammation in liver cells; decreases inflammatory markers like NLRP3, IL-1*β*, and TNF-*α*; enhances antioxidant factors such as serum SOD and liver Nrf2; increases the expression of the bile acid synthesis enzyme CYP7A1; and upregulates autophagy in liver cells. Serum nontargeted metabolomics indicated that NaP enhances anti-inflammatory and antioxidant metabolites, including proline and N-Acetyl-L-leucine, in diabetic liver injury mice. It potentially influences purine metabolism, amino acid synthesis (e.g., arginine, tryptophan, and tyrosine), and steroid hormone biosynthesis. This study indicates that NaP may serve as a preventive and therapeutic agent for diabetic liver injury.

## 1. Backgroud

Type 2 diabetes mellitus (T2DM) is a metabolic disorder marked by persistent hyperglycemia, potentially causing damage to various organs, including the liver [1, 2]. The liver is an important organ in the storage, conversion, and metabolism of glucose and lipids in the body. When liver function is impaired, both sugar and lipid metabolisms are affected. If high blood sugar and lipid accumulation exceed the liver's ability to clear them, it can result in liver damage. When liver damage occurs, the regulatory capacity of sugar and lipid metabolism will further decrease, leading to accelerated deterioration of liver function [3–5]. Currently, the main treatment for diabetes-related liver damage in clinical practice is still blood sugar control, as there is a lack of specific drugs for treating liver damage in diabetic patients [6, 7]. Existing diabetes treatment drugs lack specificity for treating liver damage in diabetic patients, and their metabolism in the liver can exacerbate liver burden and even worsen liver damage [8].

The pathogenesis of liver damage in T2DM mirrors that of nonalcoholic fatty liver disease; however, T2DM patients exhibit a higher incidence rate, ranging from 21% to 78% [9]. These patients usually have hepatic steatosis, and some patients may develop fatty liver hepatitis and cirrhosis [10]. High blood sugar–induced oxidative stress significantly contributes to liver damage in diabetes [11]. High levels of advanced glycation end products (AGEs) in the bodies of high blood sugar patients are important proinflammatory factors. AGE accumulation in organs like the liver can trigger oxidative stress and inflammation, crucial contributors to diabetic liver damage [12, 13].

AGEs are stable covalent compounds formed through nonenzymatic glycation reactions between amino groups of macromolecules like proteins, nucleic acids, or lipids and aldehyde or ketone groups of glucose or other reducing sugars [14]. Glycation reactions occur slowly and can be cleared in a timely manner in a normal body, but in a state of sustained high blood sugar, the rate of glycation reactions significantly accelerates, leading to a marked increase in the production of AGEs and their accumulation in the body, triggering various complications of diabetes, including diabetic vascular complications, neurodegenerative diseases, and cancer [15, 16]. More and more evidence shows that activation of the AGE-RAGE pathway can induce oxidative stress reactions, leading to inflammatory reactions in liver tissues, including liver cells and hepatic stellate cells [17, 18]. The liver plays a crucial role in clearing and metabolizing circulating AGEs; thus, impaired liver function can lead to increased AGE accumulation, intensifying inflammatory damage [19–21].

Sodium propionate (NaP) is a short-chain fatty acid (SCFA) found in the intestines and blood at levels comparable to butyrate (NaB), yet its anti-inflammatory effects are less studied than those of NaB. Increasing dietary fiber or intake of SCFAs seems to be beneficial in the clinical treatment of obesity, insulin resistance, and chronic inflammation induced by high-fat diets [22–24]. However, most existing studies have focused on NaB, with few studies involving NaP or other SCFAs. This study investigates NaP's protective effects against AGE-induced liver damage in Type 2 diabetic mice, focusing on its mechanisms in reducing inflammation and oxidative stress.

## 2. Materials and Methods

### 2.1. Network Pharmacology Study

The SMILES structure and 2D SDF file of propionic acid were retrieved from the PubChem database (https://pubchem.ncbi.nlm.nih.gov/) using “propionic acid” as the keyword. These files were subsequently imported into the SwissTarget Prediction (http://swisstargetprediction.ch/) and PharmMapper (https://www.lilab-ecust.cn/pharmmapper/) databases to identify potential target genes of propionic acid. Additionally, the Comparative Toxicogenomics Database (CTD) (https://ctdbase.org/) was queried using the same keyword to obtain further propionic acid-related target genes. After merging the results based on gene name and removing duplicates, the final set of propionic acid targets was established. For T2DM-associated genes, the OMIM (http://omim.org/), TTD (https://db.idrblab.net/ttd/), and GeneCards (https://www.genecards.org) databases were systematically searched using “type 2 diabetes” as the key term. The retrieved targets were consolidated, and duplicate entries were removed based on gene nomenclature to generate the T2DM disease target dataset. Target protein UniProt IDs obtained from PharmMapper were converted to standardized gene names using the UniProt database (https://www.uniprot.org/). Finally, the VennDiagram package in R Studio (https://www.rstudio.com/) was employed to generate a Venn diagram comparing propionic acid targets and T2DM-associated genes, thereby identifying potential therapeutic targets of propionic acid for T2DM treatment.

Import potential targets into the STRING database, with the species condition set to “Homo sapiens,” to construct a medium confidence PPI network with a score threshold greater than 0.4. Save the results and import them into Cytoscape 3.10.0 software for visualization. Use the MCC algorithm in the CytoHubba plugin to select the Top 10 key targets.

Upload potential targets to the DAVID database (https://david.ncifcrf.gov/) and perform GO functional enrichment and KEGG pathway analyses, using *p* < 0.05 as the threshold for significant enrichment, to identify the biological processes (BPs) and key signaling pathways through which propionic acid may exert therapeutic effects on T2DM. Utilize the ggplot2 package in R Studio to visualize the results by creating bar graphs for the Top 10 pathways in the GO analysis enrichment of BPs, cellular components (CCs), and molecular function (MF) modules, and generate both bubble and bar graphs for the Top 15 pathways in the KEGG analysis.

### 2.2. Diabetic Mouse Model Construction and Grouping

Male ICR mice (SPF-grade, 22 ± 4 g) were sourced from Jiangsu Wukong Biotechnology Co., Ltd and housed at Jiangsu University's Animal Experiment Center. The mice were categorized into four groups: normal control (NC), high-fat with high AGEs (HFD + AGEs), high-concentration NaP (NaPH), and low-concentration NaP (NaPL).

Referring to the research methods of other scholars, drugs were administered by adding NaP to the drinking water. The NaPH group and NaPL group were, respectively, added to the drinking water with a final concentration of 1% and 0.5% NaP [25, 26], while the other groups of mice drank sterile distilled water. The T2DM mouse model was constructed using the research methods of our group in the previous study [27]; detailed steps can be found in S1. At the same time, 1 mg/g of BSA-AGEs was added to the high-fat diet of mice to accelerate the accumulation of AGEs in the mice and induce liver damage [28]. BSA-AGEs were prepared by our group according to the methods reported by Komati et al. [29]; specific methods can be found in S2.

### 2.3. Construction and Grouping of HepG2 Cell Lipid Injury Model

The HepG2 cells were generously provided by Professor Hong Zhou's laboratory at the Medical School of Jiangsu University. Cells were maintained in RPMI 1640 medium with 10% fetal bovine serum (Biological Industries, Kibbutz, Israel) at 37°C and 5% CO_2_. HepG2 cells were cultured in 6-well plates at a density of 1 × 10^6^ cells per well. The cell experiment groups consist of the NC, OA, OA + AGEs, OA + AGEs + NaPL, and OA + AGEs+NaPH groups.

In this study, oleic acid (OA) was added to cell culture medium to simulate a high-fat environment. A 15-mmol/L OA solution was provided by Sangon Biotech (Shanghai, China), and a endotoxin-free AGE-BSA is provided by Biogradetech Inc. (purity greater than 95%, molecular weight of 69.3 kDa, Shanghai, China). Cells in the NaPH and NaPL groups were pretreated with 200 and 100 *μ*mol/L NaP, respectively, for 24 h prior to exposure to OA and AGEs. Referring to Song et al., cells in the OA group were exposed to 0.05 mmol/L OA for 6 h, whereas those in the OA + AGE group received a combined treatment of 0.05 mmol/L OA and 200 *μ*g/mL AGEs for the same duration [30]. The NC group cells were cultured without any treatment. Following the experiment, the cells were harvested for additional analysis.

### 2.4. The QPCR and Western Blotting Assays

Gene and protein expression levels were assessed using qPCR and Western blotting assays. See S3 for detailed experimental procedures. The expressions of LC3B and P62 in HepG2 cells were used to evaluate autophagy. CYP7A1 serves as a rate-limiting enzyme for assessing bile acid synthesis in the liver, while Nrf2 is utilized to evaluate hepatic oxidative stress levels.

### 2.5. Analysis of Liver Tissue HE Staining and HepG2 Cell Oil Red O Staining

HE staining assessed liver damage in mice, while Oil Red O staining evaluated lipid deposition in HepG2 cells. We also used the triglyceride test kits to detect the lipid content in HepG2 cells. For detailed operating steps, refer to S4.

### 2.6. Detection of Biochemical-Related Indexes in Mouse Serum

Biochemical indicators in mouse serum and triglyceride levels in HepG2 cells were analyzed using reagents from Nanjing Jiancheng Bioengineering Institute, China. Testing procedures strictly followed the reagent kit instructions. The detailed operating steps were seen in S5.

### 2.7. Nontargeted Metabolomics Analysis of Mouse Serum

To further investigate the mechanism by which NaP alleviates liver damage in diabetic mice, we selected the high concentration NaP group (1% NaP) mouse serum for nontargeted metabolomics analysis, which was completed by ekemo Tech Group Co., Ltd. (Shenzhen, China). The preprocessing and detection methods of mouse serum samples refer to the report by Feng et al. [31], and the detailed operating procedures can be found in S6. A 100-*μ*L aliquot of each sample was transferred to a 1.5-mL microcentrifuge tube, followed by the addition of 400 *μ*L of 80% methanol aqueous solution. The mixture was vortexed vigorously and incubated on ice for 5 min and then centrifuged at 15,000 × g and 4°C for 20 min. A volume of the supernatant was diluted with mass spectrometry-grade water to achieve a final methanol concentration of 53%. The solution was recentrifuged under identical conditions (15,000 × g, 4°C, 20 min), and the resulting supernatant was collected for LC-MS analysis to obtain metabolite identification and relative quantification data. Mass spectrometry data was preprocessed with Progenesis QI software (v2.1, Waters Corporation, Massachusetts, United States) and imported into SIMCA-P software (v13.0, Umetrics, Umea, Sweden) for principal component analysis (PCA) and orthogonal partial least squares discriminant analysis (OPLS-DA). Differential metabolites were identified using criteria of VIP > 1 and *p* < 0.05. Details on differential metabolites, including their names and structures, were obtained from the Human Metabolome Database (HMDB) (https://hmdb.ca). MetaboAnalyst (https://www.metaboanalyst.ca/) was used for metabolic pathway analysis of the selected biomarkers.

### 2.8. Statistical Analysis

Data analysis was conducted using SPSS 26.0, graphs were generated with Graphpad Prism 9.0, and quantitative data are presented as mean ± standard deviation. Conduct one-way ANOVA for hypothesis testing, assess intergroup variances using the LSD method, and consider statistical significance at a *p* value of less than 0.05.

## 3. Results

### 3.1. Analysis Results of Propionic Acid Network Pharmacology

**Figure 1:**
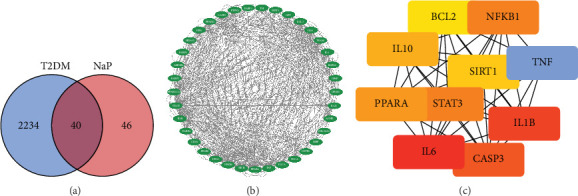
Analysis of propionic acid pharmacology network. (a) There are 40 potential target points between propionic acid and Type 2 diabetes. (b) The PPI network diagram shows the core proteins related to propionic acid treatment of Type 2 diabetes, as well as (c) the Top 10 core proteins.

Through searching the SwissTargetPrediction database, Targets SUPPRED database, and other databases, 260 potential target points were obtained after screening and removing duplicates. Through searching the Disgenet database, GeneCards database, OMIM database, and others, 2274 T2DM target points were obtained after screening and removing duplicates. Using the SRplot online analysis platform (https://www.bioinformatics.com.cn/srplot), the target points of propionic acid drugs and T2DM were inputted into a Venn diagram, resulting in 40 potential target points for propionic acid treatment of T2DM (Figure 1a).

Use Cytoscape 3.10 software to draw the PPI network diagram of propionic acid treating T2DM (Figure 1b). Use the maximal clique centrality (MCC) function in the CytoHubba plugin of Cytoscape software to select the Top 10 hub genes in the PPI network, mainly involving mitochondrial function, inflammation, etc., including BCL-2, NF-*κ*B, IL-10, SIRT1, TNF, PPAR*α*, STAT3, IL-1*β*, IL-6, and CASP3 (Figure 1c).

**Figure 2:**
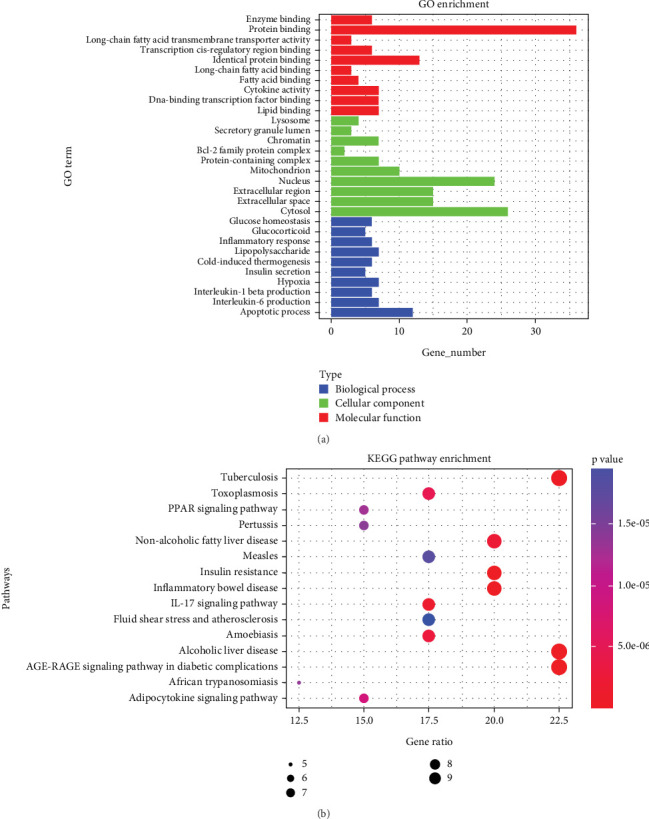
(a) The GO analysis shows the gene and protein functional classifications involved in the treatment of T2DM with propionic acid. (b) The KEGG analysis shows the metabolic pathways involved in the treatment of T2DM with propionic acid.

The GO analysis indicates that BPs primarily encompass enzyme binding, protein binding, long-chain fatty acid transmembrane transporter activity, and fatty acid binding; the entries related to CCs mainly involve lysosome, secretory granule lumen, chromatin, Bcl-2 family protein complex, protein-containing complex, and mitochondrion; the entries related to MFs mainly involve glucose homeostasis, glucocorticoid, inflammatory response, insulin secretion, IL-1*β* production, IL-6 production, etc. According to the rule *p* < 0.05, the Top 10 pathways were visualized for analysis (Figure 2a). The KEGG analysis results, depicted in Figure 2b, primarily involve nonalcoholic fatty liver disease, insulin resistance, the IL-17 signaling pathway, and AGE-RAGE signaling in diabetic complications.

### 3.2. NaP Significantly Reduced Lipid Deposition and Triglyceride Content in HepG2 Cells

**Figure 3:**
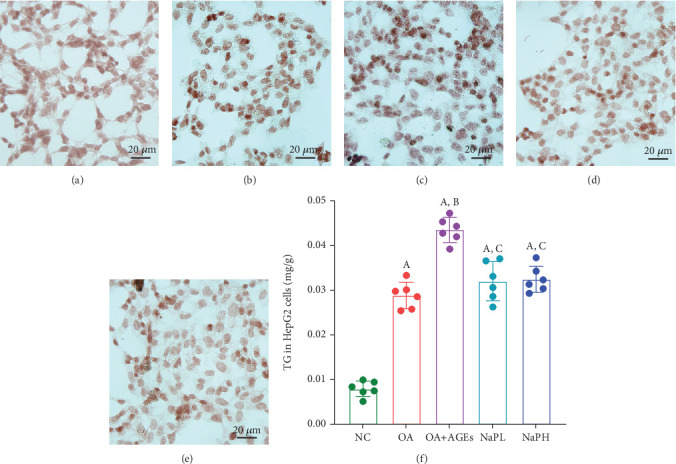
(a–e) The lipids in HepG2 cells were observed by Oil Red O staining, and the intracellular lipids showed red granules. (f) The TG detection kit was used to detect the intracellular TG content. A: compared with the NC group, *p* < 0.05; B: compared with the OA group, *p* < 0.05; C: compared with the OA + AGE group, *p* < 0.05. *n* = 6.

After staining with Oil Red O, the lipids of HepG2 cells became red granules (Figure 3). The findings indicate an increased presence of red granules in the OA group cells compared to the NC group (Figure 3b). This prominence is further enhanced in the OA + AGE group following the addition of AGEs (Figure 3c). After treatment with high and low concentrations of NaP, the red lipid granules in the cells significantly decreased (Figure 3d,e).

Further use of the TG test kit to detect intracellular lipid concentration, the experimental results are consistent with Oil Red O staining results (Figure 3f). The intracellular TG content was significantly elevated in both the OA and OA + AGE groups compared to the NC group, with the OA + AGE group showing a significantly higher level than the OA group (*p* < 0.05). Following NaP treatment, the intracellular TG content showed no significant difference between the NaPL and NaPH groups (*p* > 0.05), was significantly lower than the OA + AGE group (*p* < 0.05), and was comparable to the OA group (*p* > 0.05).

### 3.3. NaP Upregulates Autophagy Levels and Antioxidant Stress Capacity in HepG2 Cells and Enhances Bile Acid Synthesis Capacity

**Figure 4:**
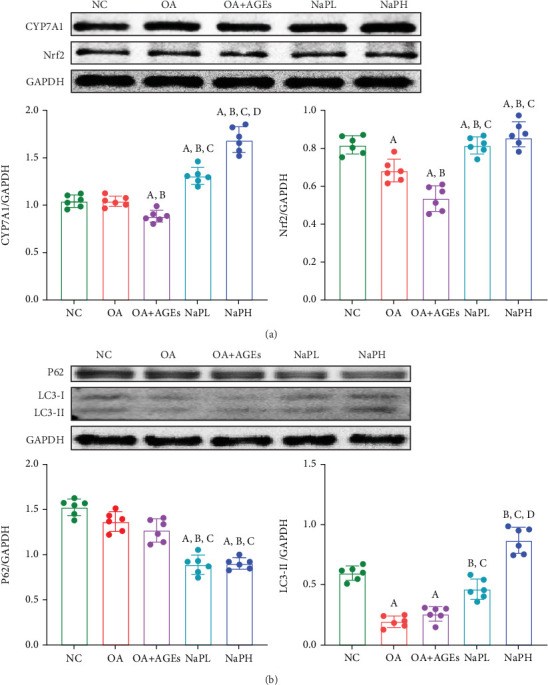
Western blotting was used to detect the expression levels of (a) bile acid synthesis enzyme CYP7A1 and oxidative stress-related protein Nrf2 and (b) autophagy-related proteins P62 and LC3 in HepG2 cells. A: compared with the NC group, *p* < 0.05; B: compared with the OA group, *p* < 0.05; C: compared with the OA + AGE group, *p* < 0.05; D: compared with the NaPL group, *p* < 0.05. *n* = 6.

CYP7A1 is a crucial enzyme that regulates the rate of bile acid production in the liver within the classic cholesterol synthesis pathway. CYP7A1 expression in HepG2 cells significantly decreased following OA + AGE treatment; however, pretreatment with both high and low concentrations of NaP significantly increased CYP7A1 expression compared to the OA + AGE group (*p* < 0.05) (Figure 4a), suggesting improved bile acid synthesis in liver cells. This result suggests that liver cells will consume more lipids for bile acid synthesis, thereby reducing lipid deposition in the liver.

Nrf2 is mainly responsible for enhancing the antioxidant stress response and protecting cells from oxidative stress damage. After treatment with OA and AGEs, the expression of Nrf2 in HepG2 cells was significantly decreased (*p* < 0.05). Pretreatment with both high and low concentrations of NaP significantly increased Nrf2 expression compared to the OA and OA + AGE groups (*p* < 0.05) (Figure 4a), suggesting an inhibition of oxidative stress in liver cells.

Both P62 and LC3 are proteins related to cell autophagy. During autophagy, the expression of P62 decreases, while the expression of LC3-II increases. Treatment with OA and AGEs significantly decreased autophagy levels in HepG2 cells, whereas pretreatment with both high and low concentrations of NaP significantly increased autophagy levels compared to the OA and OA + AGE groups (*p* < 0.05) (Figure 4b).

### 3.4. Effects of NaP on Body Weight and Serum Biochemical Indices

**Figure 5:**
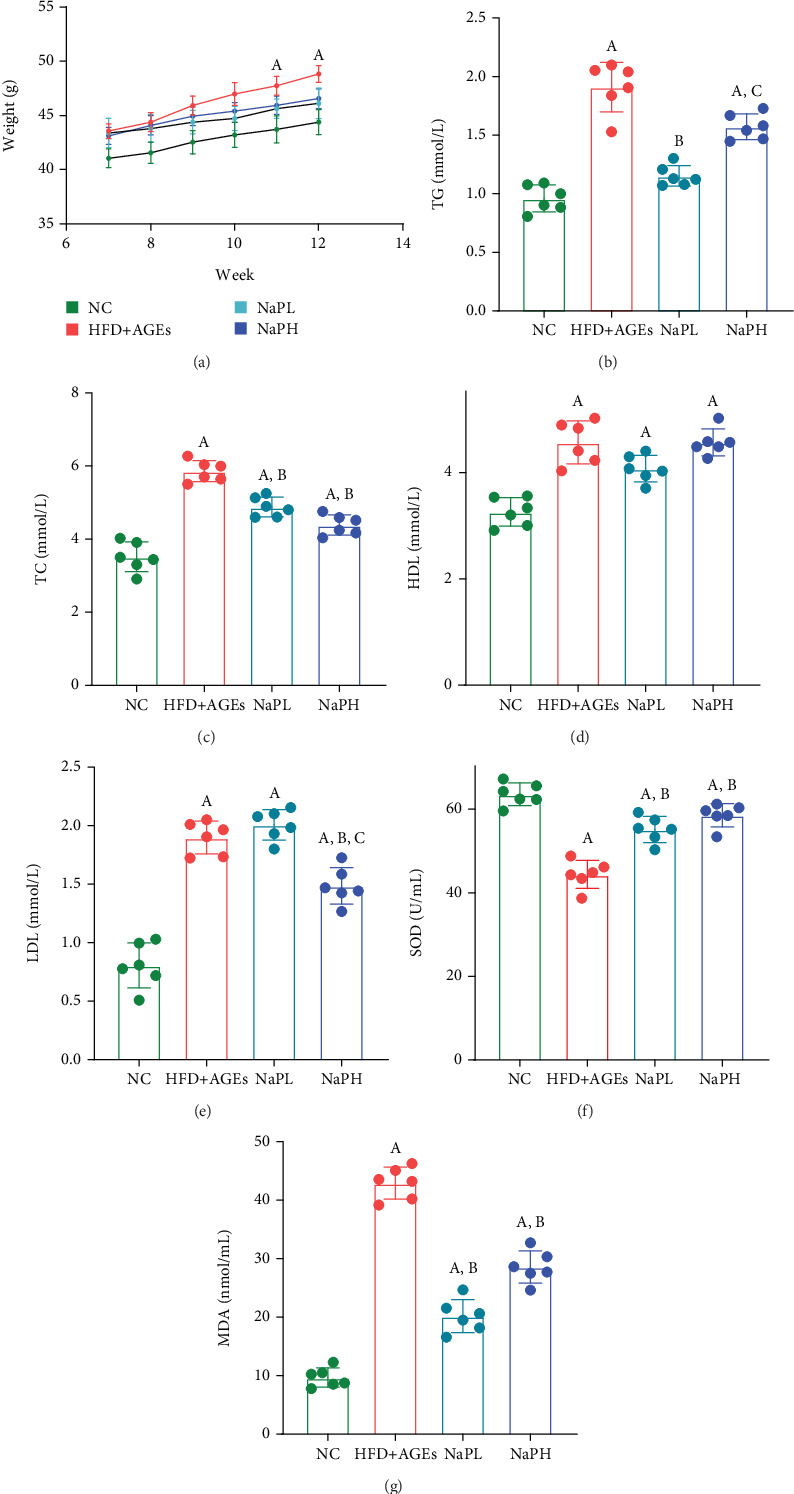
NaP can significantly inhibit the levels of (a) body weight, (b) TG, (c) TC, and (g) MDA and increase the (f) SOD level in HFD + AGE group mice; high-level NaP can lower the level of (d) LDL but has no significant effect on (e) HDL level. A: compared with the NC group, *p* < 0.05; B: compared with the HFD + AGE group, *p* < 0.05; C: compared with the NaPL group, *p* < 0.05. *n* = 6.

From the seventh week, mice in the HFD + AGE group exhibited a rapid increase in body weight, becoming significantly higher than the other three groups during the 11th and 12th weeks (*p* < 0.05) (Figure 5a).

In the HFD + AGE groups, blood lipid indicators TG, TC, HDL, LDL, and MDA were significantly elevated compared to the NC group, whereas the oxidative stress indicator SOD was significantly reduced (*p* < 0.05). Following treatment with both high and low concentrations of NaP, there was a significant reduction in TG, TC, LDL, and MDA levels, alongside a significant increase in SOD, compared to the HFD + AGE group (*p* < 0.05) (Figures 5b, 5c, 5d, 5e, 5f, and 5g).

### 3.5. NaP Inhibits Liver Inflammation and Oxidative Stress Reactions in Mice and Upregulates Bile Acid Synthesis

**Figure 6:**
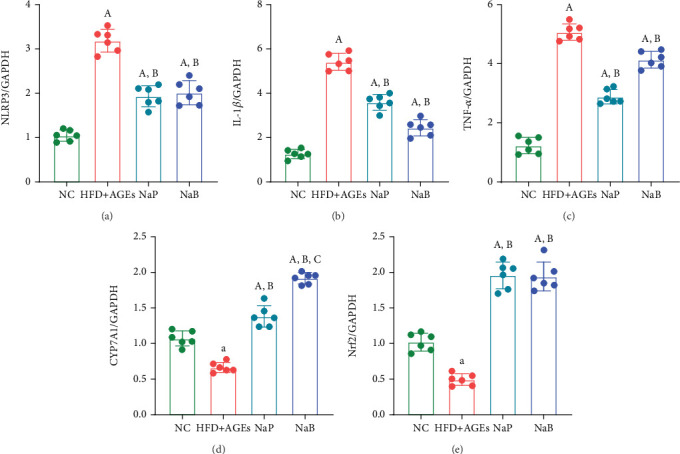
NaP inhibited the expression of inflammatory factors (a) NLRP3, (b) IL-1*β*, and (c) TNF-*α* in the liver of mice fed a high-fat-and-AGEs diet and increased the expression of (e) the antioxidant stress factor Nrf2 and (d) the bile acid synthesis enzyme CYP7A1. A: compared with the NC group, *p* < 0.05; B: compared with the HFD + AGE group, *p* < 0.05; C: compared with the NaPL group, *p* < 0.05. *n* = 6.

A diet high in fats and AGEs significantly elevated the expression of inflammatory markers NLRP3, IL-1*β*, and TNF-*α* in mouse liver, while reducing the expression of the antioxidant factor Nrf2 and the bile acid synthesis enzyme CYP7A1 (*p* < 0.05). NaP, at both high and low concentrations, effectively reduces oxidative stress and inflammation caused by high-fat and AGEs, as indicated by decreased levels of inflammatory markers NLRP3, IL-1*β*, and TNF-*α*, and increased levels of the antioxidant factor Nrf2 in mouse liver compared to the HFD + AGE group (*p* < 0.05) (Figure 6). The effects of high and low NaP concentrations did not differ significantly (*p* > 0.05). The hepatic expression of the crucial bile acid synthesis enzyme CYP7A1 was significantly elevated (*p* < 0.05), indicating that NaP enhances bile acid production, which in turn facilitates cholesterol consumption and reduces its accumulation in the liver.

### 3.6. NaP Reduces Liver Damage in High-Fat and AGE Diet Mice

**Figure 7:**
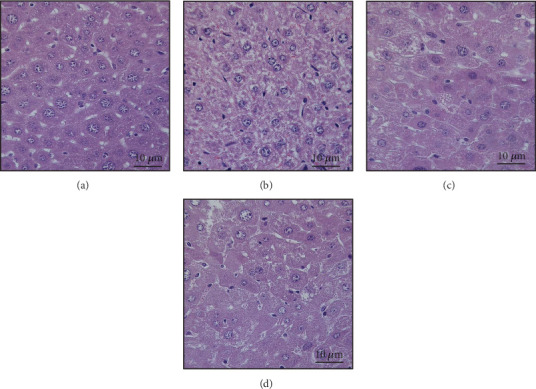
HE staining showed that (a) there were no obvious pathological changes in the liver of mice in the NC group, while (b) the liver in the HFD + AGE group showed significant fatty changes. (c, d) The liver damage was significantly reduced after intervention with high and low concentrations of NaP.

Staining shows that the liver lobule structure of the NC group mice is intact, with liver cells arranged neatly, rare fat degeneration, clear cell structure and rich cytoplasm, cell nuclei located in the center of the cell, and no inflammatory cell aggregation in the liver lobule and around blood vessels (Figure 7a). The HFD + AGE group showed obvious fatty liver-like lesions, with significant fat degeneration in liver tissue. Some liver cells are filled with large lipid droplets vacuoles in the cytoplasm, and the cell nuclei have been pushed to one side by the lipid droplets (Figure 7b). In diabetic mice with liver damage, treatment with both high and low concentrations of NaP led to a reduction in hepatic tissue steatosis and a significant decrease in the number and size of cytoplasmic lipid droplets compared to the HFD + AGE group (Figure 7c,d).

### 3.7. Nap Enhances Serum Metabolomics in Mice With Diabetic Liver Injury

**Figure 8:**
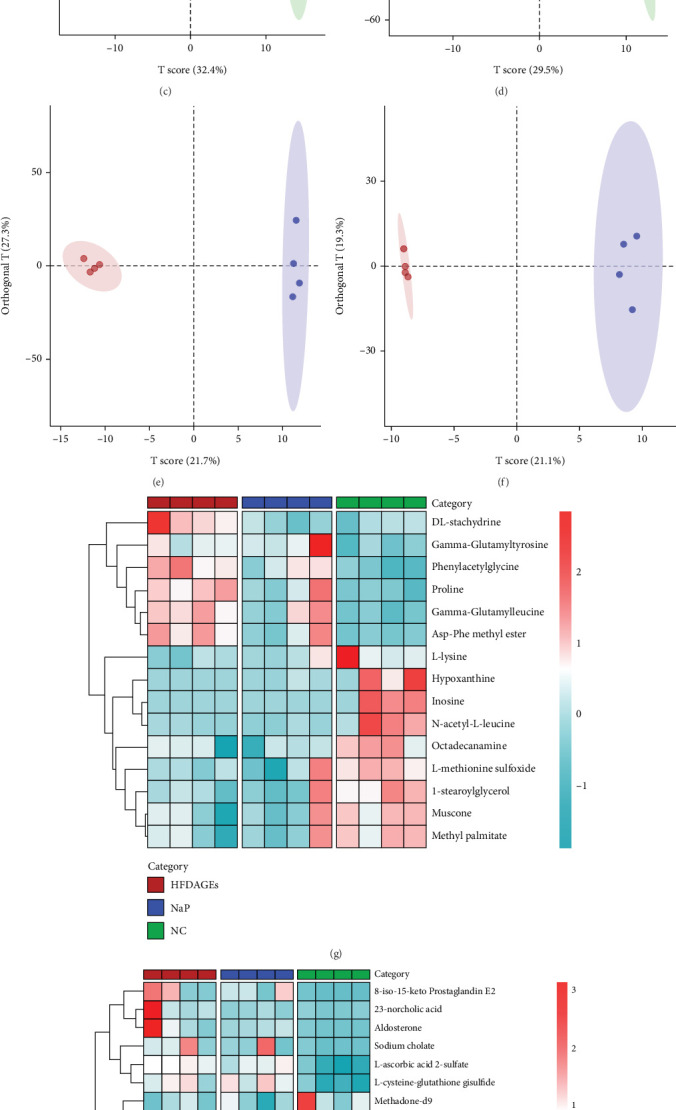
The effect of NaP on the serum metabolomics of diabetic liver-damaged mice (n = 4). (a, b) The PCA analysis results showed that in both ESI+ and ESI- modes, the samples of the HFD + AGE group and NaP group were significantly separated from the NC group, but the samples of the HFD + AGE group and NaP group were not completely separated. (c–h) Through further OPLS-DA analysis, differential metabolites were selected based on VIP > 1 and *p* < 0.05 rules and visualized as a heatmap. (i, j) The differential metabolites were analyzed through the MetaboAnalyst website to obtain relevant metabolic pathways (i, j). In the ESI+ mode, the main metabolic pathways involved include (1) purine metabolism, (2) amino sugar and nucleotide sugar metabolism, (3) biotin metabolism, arginine biosynthesis, (5) retinol metabolism, (6) starch and sucrose metabolism, (7) interconversion of pentose and glucuronate metabolism, and (8) steroid hormone biosynthesis. In the ESI- mode, the main metabolic pathways involved include (1) riboflavin metabolism, (2) purine metabolism, (3) histidine metabolism, (4) terpenoid backbone biosynthesis, (5)pyrimidine metabolism, (6) tryptophan metabolism, (7) tyrosine metabolism, and (8) steroid biosynthesis.

In order to further study the mechanism of NaP in improving diabetes liver damage, we used nontargeted metabolomics analysis to study the effects of high concentration NaP (1%) on the serum metabolomics of diabetes liver damage mice. PCA results showed that in both ESI^+^ and ESI^−^ modes, the HFD + AGE group and the NaP group were significantly separated from the NC group, but the HFD + AGE group and the NaP group were not completely separated (Figure 8a,b). This result indicates the successful construction of the diabetes liver damage model. Differential metabolites were further selected using the OPLS-DA model, applying criteria of VIP > 1 and *p* < 0.05, and results were visualized in a heatmap (Figures 8c, 8d, 8e, 8f, 8g, and 8h).

The heatmap of differential metabolites showed significant changes in the production of metabolites related to anti-inflammatory and antioxidant stress, including proline, N-Acetyl-L-leucine, hypoxanthine, phenylacetylglycine, and allantoin (Tables 1 and 2). The metabolites primarily participated in purine, amino sugar, and nucleotide sugar metabolism; biotin, arginine, histidine, and tryptophan biosynthesis; riboflavin and retinol metabolism; starch and sucrose metabolism; pentose and glucuronic acid ester interconversion; and steroid biosynthesis (Figure 8i,j).

## 4. Discussion

Accumulating evidence highlights the regulatory roles of SCFAs in hepatic pathologies, particularly NaB and acetate (NaAc), which have been extensively studied in metabolic dysfunction-associated steatotic liver disease (MASLD) and cirrhosis. NaB primarily produced by *Clostridium* spp., exerts hepatoprotective effects through multiple mechanisms: (i) As a histone deacetylase (HDAC) inhibitor, NaB enhances hepatic AMPK phosphorylation, thereby suppressing de novo lipogenesis (DNL) via downregulating SREBP-1c and ACC1 in HFD-fed mice [32]; (ii) it ameliorates gut barrier dysfunction by upregulating tight junction proteins (e.g., ZO-1 and occludin), thus reducing portal venous endotoxin influx and subsequent Kupffer cell activation [33]; (iii) clinical trials suggest that oral NaB supplementation (4 g/day) significantly lowers ALT levels and hepatic steatosis scores in NAFLD patients [34].

In contrast, NaAc—the most abundant circulating SCFA—demonstrates dual effects on liver metabolism. While NaAc administration promotes DNL through ACLY-dependent acetyl-CoA synthesis in hepatocytes [35], chronic exposure improves insulin sensitivity by activating GPR43 in HFD-induced NAFLD models [36]. Notably, NaAc-NaB cosupplementation synergistically reduces hepatic TNF-*α* and IL-6 expression via NF-*κ*B/NLRP3 inflammasome inhibition, suggesting combinatorial SCFA therapy may optimize efficacy [37].

NaP in the human body mainly comes from the fermentation of dietary fiber that cannot be digested by oneself in the colon by certain anaerobic bacteria, which belongs to a type of SCFAs [38]. However, the beneficial physiological functions of NaP have long been underestimated [39, 40]. Recent studies have demonstrated that NaP can lower fatty acid levels in the liver and plasma, curb appetite, reduce chronic inflammation, and potentially enhance tissue insulin sensitivity. Increasing the intake of exogenous NaP or the production of NaP in the intestine is believed to be beneficial for preventing obesity and T2DM [22, 41].

Diabetes patients are prone to liver and gallbladder diseases, such as fatty liver disease and cirrhosis. Cirrhosis is a leading cause of mortality among patients with diabetes [42]. In diabetes patients, lipid accumulation in liver cells and oxidative stress from reactive oxygen species formation can elevate the risk of liver cancer. Identifying novel approaches for preventing and treating liver damage in diabetic patients is highly significant.

Through network pharmacology analysis, it was found that NaP can act on various oxidative stress and inflammatory factors of T2DM, including NF-*κ*B, TNF, IL-1*β*, SIRT1, and IL-6. NaP plays a role in regulating insulin resistance, mitochondrial function, and the production of IL-1*β*, IL-6, and IL-17 in T2DM. Its anti-inflammatory and antioxidant capabilities are closely related to its therapeutic effect on diabetic liver damage. To confirm the results of network pharmacology analysis, we further constructed HepG2 cell models and T2DM mouse models for research.

AGEs are a crucial factor in the development of complications in diabetic patients [15, 43, 44]. High levels of AGEs can worsen insulin resistance, leading to liver and kidney damage as well as systemic microvascular damage [14, 15, 45]. This study found that a high-fat and AGEs diet can induce inflammation and oxidative stress in the liver of mice, leading to significant liver damage. It also inhibits the activity of the rate-limiting enzyme CYP7A1 in bile acid synthesis in the liver, which may further inhibit the use of cholesterol to synthesize bile acids, causing massive lipid deposition in liver cells. HepG2 cell experiments further show that in comparison to a high OA environment alone, a high OA-and-AGEs environment has a stronger ability to induce lipid deposition and oxidative stress in liver cells, and significantly inhibits the expression of bile acid synthesis enzyme CYP7A1 and the autophagy ability of liver cells.

In mammals, bile acid biosynthesis in the liver initiates with the 7*α*-hydroxylation of cholesterol [46]. Under typical circumstances, the classic pathway generates at least half of the bile acids in humans [47]. Research indicates that CYP7A1 is a crucial rate-limiting enzyme in bile acid synthesis, with increased hepatic expression accelerating bile acid production and lipid clearance, including cholesterol, in the liver [48, 49]. Our research has found that NaP can upregulate the expression of CYP7A1 in the liver at both systemic and extracellular levels, which will help in clearing excess lipids from the liver. The reason for liver damage in diabetes is that the liver takes up more fatty acids than it needs for its own metabolism and converts the excess fatty acids into triglycerides stored in the liver [49–51]. Excessive lipids in the liver can lead to hepatic steatosis, and the production of large amounts of reactive oxygen species and lipid peroxides. Damage to the liver caused by reactive oxygen species and lipid peroxides can progress to fatty liver disease, fibrosis, and cirrhosis [52]. Therefore, accelerating the clearance of lipids in the liver with NaP will help alleviate liver damage in diabetes.

Autophagy also helps liver cells reduce inflammation damage and lipid deposition. Autophagy is a distinct cell death process in mammalian liver cells, used to remove damaged mitochondria and excess fat, thereby preserving cellular homeostasis [53]. Research by Xie et al. has demonstrated the significance of autophagy in the development of NAFLD, with blocked autophagy in liver cells leading to increased fat accumulation and fibrosis [54]. Our study demonstrated that NaP effectively decreases lipid accumulation and inflammation in liver cells caused by a high-fat diet and AGEs, while enhancing autophagy levels. We proposed that the enhancement of autophagy by NaP is significantly linked to its capacity to ameliorate diabetic liver injury. In order to further explore the mechanism of NaP in alleviating liver damage in diabetic mice, we used nontargeted metabolomics technology to analyze the effect of NaP on the serum metabolomics of diabetic liver-damaged mice.

Our studies have shown that NaP can both affect pyrimidine metabolism, steroid biosynthesis, and tryptophan metabolism in diabetic liver injury mice. Chronic low-grade inflammation is central to diabetes pathogenesis, with tryptophan metabolism serving as a therapeutic target to mitigate this inflammation [55]. Tryptophan metabolism produces metabolites like kynurenine, xanthurenic acid, indole-3-acetic acid, and serotonin/melatonin via its three primary pathways: the kynurenine, indole, and serotonin/melatonin pathways [56, 57]. Indoleamine 2,3-dioxygenase (IDO) regulates the kynurenine pathway and is upregulated by proinflammatory molecules like TNF-*α*, IL-6, and LPS [58]. Increased IDO activity correlates with heightened inflammation and fibrosis in NAFLD, alongside elevated blood glucose, obesity, and atherosclerosis [59–61]. Conversely, elevated levels of metabolites from the gut microbiota-regulated indole pathway have therapeutic effects on NAFLD [62]. Upon reaching the colon, dietary tryptophan is metabolized by gut microbiota into various indoles, such as indole-3-acetic acid ester (IA), indole-3-propionic acid ester (IPA), and skatole (3-methylindole). Receptors such as the aryl hydrocarbon receptor (AhR) and pregnane X receptor (PXR) play a crucial role in linking tryptophan metabolites from gut microbiota to inflammation, acting as key regulatory mediators of the gut–liver axis [63, 64]. These intracellular receptors can suppress gluconeogenesis and lipid metabolism while reducing inflammation by inhibiting the NF-*κ*B pathway [55].

Although our research results indicate that NaP has a protective effect against AGEs and lipid-induced diabetic liver injury, key translational issues still require further investigation. In the next phase of research, we will conduct clinical trials to explore the impact of NaP on gut microbiota and assess its protective effects on patients with diabetic liver disease.

## 5. Conclusion

AGEs can exacerbate oxidative stress and lipid deposition in liver cells, which is an important factor in worsening liver damage in diabetic patients. NaP can alleviate liver damage in diabetic patients, possibly by upregulating liver cell autophagy to inhibit lipid deposition, inflammation, and oxidative stress in the liver. Further serum metabolomics results suggest that NaP can affect metabolic pathways, such as tryptophan in diabetic liver damage mice, and inhibit liver inflammation damage by intervening in tryptophan metabolism pathways.

## Figures and Tables

**Table 1:** Differential metabolic products in the serum of mice from the HFD + AGE group and the NaP group (ESI^+^).

**No.**	**Metabolite**	**Formula**	**HMDB**	**m**/**z**	**Retention time (min)**	**Which-max**
1	1-Stearoylglycerol	C_21_H_42_O_4_	HMDB0244009	381.29764	10.654	↓
2	L-methionine sulfoxide	C_5_H_11_NO_3_S	HMDB0250776	166.05344	2.046	↓
3	Proline	C_5_H_9_NO_2_	HMDB0003411	116.07076	1.414	↑
4	N-acetyl-L-leucine	C_8_H_15_NO_3_	HMDB0011756	174.11276	5.664	↑
5	Hypoxanthine	C_5_H_4_N_4_O	HMDB0000157	137.04598	2.031	↓
6	Inosine	C_10_H_12_N_4_O_5_	HMDB0000195	269.08816	3.463	↓
7	Phenylacetylglycine	C_10_H_11_NO_3_	HMDB0000821	194.08146	5.524	↑
10	Muscone	C_16_H_30_O	HMDB0034181	239.23706	9.833	↓
11	L-lysine	C_6_H_14_N_2_O_2_	HMDB0000182	147.11296	1.321	↓
12	Methyl palmitate	C_17_H_34_O_2_	HMDB0061859	271.2633	9.832	↓
13	Gamma-glutamylleucine	C_11_H_20_N_2_O_5_	HMDB0011171	261.14479	5.386	↑
15	Gamma-glutamyltyrosine	C_14_H_18_N_2_O_6_	HMDB0011741	311.12399	4.985	↓

**Table 2:** Differential metabolic products in the serum of mice from the HFD + AGE group and the NaP group (ESI^−^).

**No.**	**Metabolite**	**Formula**	**HMDB**	**m**/**z**	**Retention time (min)**	**Which-max**
1	Indole-3-lactic acid	C_11_H_11_NO_3_	HMDB0000671	204.06608	5.654	↓
2	Adrenic acid	C_22_H_36_O_2_	HMDB0002226	331.26416	10.378	↓
3	1-Palmitoyl-Sn-glycero-3-phosphocholine	C_24_H_50_NO_7_P	HMDB0010382	494.325	9.611	↑
4	Aldosterone	C_21_H_28_O_5_	HMDB0000037	359.18664	6.268	↑
5	Capryloylglycine	C_10_H_19_NO_3_	HMDB0000832	200.12874	6.185	↑
6	Indole-3-pyruvic acid	C_11_H_9_NO_3_	HMDB0060484	202.05028	5.55	↓
7	L-ascorbic acid 2-sulfate	C_6_H_8_O_9_S	HMDB0060649	254.98145	1.401	↑
8	Imidazoleacetic acid	C_5_H_6_N_2_O_2_	HMDB0002024	125.03454	4.896	↓
9	Allantoin	C_4_H_6_N_4_O_3_	HMDB0000462	157.03577	1.35	↓

## Data Availability

The data that support the findings of this study are available from the corresponding authors upon reasonable request.

## Supporting Information

Supporting Information Additional supporting information can be found online in the Supporting Information section. S1. Experimental procedure of mouse diabetic liver injury model. S2. BSA-AGEs preparation. S3. Experimental procedure of the qPCR assay and the Western blotting assay. S4. HepG2 cell oil red O staining. S5. Detection of biochemical-related indexes in mouse serum. S6. Serum nontargeted metabolomics analysis.
