# Supplementary material for: Network Pharmacology and Serum Nontargeted Metabolomics Reveal the Protective Effects of Propionate Against Liver Damage Induced by a High-Fat and AGE-Rich Diet in Diabetic Mice
Source: J Diabetes Res. 2025 Jul 2;2025:3955893. doi: 10.1155/jdr/3955893 (PMC12240661; doi:10.1155/jdr/3955893)
Supplement: Supporting Information — Additional supporting information can be found online in the Supporting Information section. S1. Experimental procedure of mouse diabetic liver injury model. S2. BSA-AGEs preparation. S3. Experimental procedure of the qPCR assay and the Western blotting assay. S4. HepG2 cell oil red O staining. S5. Detection of biochemical-related indexes in mouse serum. S6. Serum nontargeted metabolomics analysis. [file 3955893.f1.doc]

**Supplement**

*S1.* *Experimental Procedure of Mouse Diabetic Liver Injury Model.* The NC group mice were fed a regular diet, while HFD+AGEs group, NaPL group, and NaPH group mice were fed a diet with 40% fat energy content + AGEs (1 mg/g). The NaPH group and NaPL group were respectively added to the drinking water with a final concentration of 1% and 0.5% NaP. The NC group and HFD+AGEs group mice were given sterile distilled water. The weight of the mice in each group were measured weekly, and the experiment was ended after 12 weeks of continuous intervene. At the end of the experiment, mice were euthanized by intraperitoneal injection of Urethan (Sigma-Aldrich, St. Louis, MO, USA, 700 mg/kg). The serum was used for untargeted metabolomics analysis and other tests.

*S2. BSA-AGEs Preparation.* The method of BSA-AGEs preparation was as follows: dissolve bovine serum albumin (BSA) and glyoxal in 0.1 mol/L PBS buffer (pH 7.4), with a final concentration of 0.1 mmol/L for BSA and 100 mmol/L for glyoxal. After mixing the above solution well and sealing it from light, it is placed in a 37 °C constant temperature box for 72 hours to generate AGEs. After the incubation, the solution was dialyzed in 0.1 mol/L ammonium bicarbonate buffer (pH 8.0) to remove unbound glyoxal and other small molecules. After dialysis, the BSA-AGEs was freeze-dried to prepare a powder and handed over to Future Biotech (Beijing, China) to prepare high AGEs feed, with an addition of 1 mg/g of AGEs. The content of various nutrients in the feed can be found in TABLE 1.

TABLE 1. Nutrient composition table of mouse high fat feed (40%)

| Product | gm | kcal(%) |
| --- | --- | --- |
| Protein | 23 | 20 |
| Carbohydrate | 46 | 40 |
| Fat | 20 | 40 |
| Total |  | 100 |
| kcal/gm | 4.55 | |

*S3. Experimental Procedure of the QPCR Assay and the Western Blottng Assay.* The total RNA was extracted from mouse liver tissue using the total RNA extraction kit (Vazyme Biotechnology, Nanjing, China) and reverse transcribed into cDNA (Vazyme Biotechnology, Nanjing, China) for qRT-PCR analysis (CFX96, Bio-Rad, Hercules, CA, USA). The PCR primers were synthesized by GENEWIZ company in Suzhou, China, and the primer sequences can be found in TABLE 2. The total PCR reaction system included 10 μL of SYBR Green Master premix (Vazyme Biotechnology, Nanjing, China), 0.4 μL (10 μmol/L) of forward and reverse primers, and 2 μL of cDNA template. The reaction process included an initial denaturation at 95°C for 5 min, denaturation at 95°C for 3 s, annealing at 58°C for 20 s, extension at 72°C for 30 s, for a total of 40 cycles. The β-actin gene was used as an internal reference, and relative gene expression was calculated using the 2^-ΔΔCt^ method. The sequences of the PCR primers can be found in TABLE 2.

TABLE 2. PCR primer sequence

| genes | Primer sequence |
| --- | --- |
| *β-actin* | F: AAGCTGTGCTATGTTGCTCTA  R: GTTTCATGGATGCCACAGGA |
| *TNF-α* | F: AACTCCAGGCGGTGCCTATG  R: TCCAGCTGCTCCTCCACTTG |
| *IL-1β* | F: CCTGTCCTGCGTGTTGAAAGA  R: GGGAACTGGGCAGACTCAAA |
| NLRP3 | F：ATTACCCGCCCGAGAAAGG  R：TCGCAGCAAAGATCCACACAG |
| *Caspase-1* | F: GCACAAGACCTCTGACAGCA  R: TTGGGCAGTTCTTGGTATTC |

Cellular total protein extraction was performed as follows. Collect all cells and wash twice with pre-cooled phosphate-buffered saline (PBS) to remove residual cell culture medium. Add RIPA lysis buffer (Beyotime Biotechnology, Shanghai, China) to lyse cells and release total proteins, followed by centrifugation at 13500×g for 20 minutes to collect the supernatant for subsequent Western blotting analysis. The extracted cellular proteins were mixed with an appropriate amount of 5× SDS-PAGE loading buffer and boiled for 10 minutes to fully denature them for SDS-PAGE electrophoresis. After electrophoresis, the proteins were transferred to a PVDF membrane (0.45 μm, Thermo Fisher Scientific, Waltham, MA, USA). After transfer, the PVDF membrane was blocked with 5% skim milk at room temperature for 1 hour. Antibodies against LC3Ⅱ (1:500, Affinity, Wuhan, China), P62 (1:1000, Affinity, Wuhan, China), CYP7A1 (1:1000, Affinity, Wuhan, China), Nrf2 (1:1000, Affinity, Wuhan, China), and GAPDH (1:10 000, Boster, Wuhan, China) were diluted in TBST buffer and incubated overnight at 4 °C. After incubation, the PVDF membrane was washed three times with TBST buffer to remove unbound antibodies, followed by incubation with goat anti-rabbit IgG-HRP (1:8000, Boster, Wuhan, China) at room temperature for 1 hour. The expression of target proteins was analyzed and quantified using the ECL chemiluminescence kit (Millipore, Boston, MA, USA) and Image J software (NCBI, Bethesda, MD, USA).

*S4. HepG2 Cell Oil Red O Staining.* The operation steps of Oil Red O staining for cells are as follows. Wash HepG2 cells with sterile PBS buffer (pH 7.4) to completely remove residual culture medium. Add an appropriate amount of 10% formalin solution to fix the cells, then add 0.5% Oil Red O working solution to stain for 2 hours. Wash the cells with sterile PBS buffer to remove residual staining solution. Observe the lipid deposition in the cells under a microscope. Additionally, collect the cells in a 6-well cell culture plate, add 200 μL of isopropanol to the cells, and measure the absorbance value (A) at 510 nm of the solution after complete dissolution .

*S5. Detection of Biochemical Related Indexes in Mouse Serum.* The mouse serum biochemical indicators were analyzed using reagents obtained from Nanjing Jiancheng Bioengineering Institute in Nanjing, China. These included tests for triglycerides (TG, A110-1-1), total cholesterol (TC, A111-1-1), low-density lipoprotein cholesterol (LDL-C, A113-1-1), high-density lipoprotein cholesterol (HDL-C, A112-1-1), total superoxide dismutase (SOD, A001-1-2), and malondialdehyde (MDA, A003-1-2). The testing procedures were conducted in strict accordance with the instructions provided with the reagent kits.

Triglycerides in HepG2 cells were measured using the triglyceride assay kit (F001-1-1) from Nanjing Jiancheng Bioengineering Institute in Nanjing, China. Following the experiment, cells were treated with trypsin digestion solution, collected by centrifugation at 1000×g for 3 minutes, and washed with sterile PBS buffer to remove any remaining cell culture medium. The cells were then lysed with 500 μL of isopropanol on ice for 2 hours, followed by centrifugation at 4 ℃, 16000×g for 10 minutes. The resulting supernatant was vacuum-dried and dissolved in 20 μL of isopropanol for triglyceride concentration measurement. The total protein concentration in the samples was determined using the Lowry method (Beyotime, ST2070, Shanghai, China). The results are presented as the amount of triglycerides (mg/g) per gram of total cellular protein.

*S6. Serum Non-targeted Metabolomics Analysis.* UPLC-QTof-MS/MS technology was used to analyze the non-targeted metabolomics of mouse serum, and LC-MS/MS technology was used to analyze the concentrations of propionate and butyrate in mouse feces. The analysis of mouse serum non-targeted metabolomics was completed by Shenzhen Wekemo Co., Ltd (Shenzhen, China). A 100 μL aliquot of each sample was transferred to a 1.5 mL microcentrifuge tube, followed by the addition of 400 μL of 80% methanol aqueous solution. The mixture was vortexed vigorously and incubated on ice for 5 min, then centrifuged at 15,000 × g and 4°C for 20 min. A volume of the supernatant was diluted with mass spectrometry-grade water to achieve a final methanol concentration of 53%. The solution was recentrifuged under identical conditions (15,000 × g, 4°C, 20 min), and the resulting supernatant was collected for LC-MS analysis to obtain metabolite identification and relative quantification data. Mass spectrometry data was imported into SIMCA-P software (v13.0, Umetrics, Umea, Sweden) for principal component analysis (PCA) and orthogonal partial least squares discriminant analysis (OPLS-DA). Differential metabolites were identified using criteria of VIP>1 and *P*<0.05. MetaboAnalyst (https://www.metaboanalyst.ca/) was used for metabolic pathway analysis of the selected biomarkers.Mass spectrometry data was pre-processed with Progenesis QI software (v2.1, Waters Corporation, MA, USA) and imported into SIMCA-P software (v13.0, Umetrics, Umea, Sweden) for principal component analysis (PCA) and orthogonal partial least squares discriminant analysis (OPLS-DA). Differential metabolites were identified using criteria of VIP>1 and *P*<0.05. Details on differential metabolites, including their names and structures, were obtained from the Human Metabolome Database (HMDB, https://hmdb.ca). MetaboAnalyst (https://www.metaboanalyst.ca/) was used for metabolic pathway analysis of the selected biomarkers.

.

Conflict of interest statement for production

The authors declare that they have no conflicts of interest.

Funding statement for production

This work was supported by Zhenjiang Key Project-Social Development (SH2023073), Jiangsu Province Traditional Chinese Medicine Science and Technology Development Program (MS2022126).
